# ‘For the love of God, just refer me’: a co-produced qualitative study of the experiences of people with Tourette Syndrome and tic disorders accessing healthcare services in the UK

**DOI:** 10.1136/bmjopen-2024-098306

**Published:** 2025-09-05

**Authors:** Camilla May Babbage, E Bethan Davies, Daniel P Jones, Paul Stevenson, Jennifer Salvage, Seonaid Anderson, Emma McNally, Madeleine J Groom

**Affiliations:** 1 NIHR MindTech Medtech Co-operative, Institute of Mental Health, University of Nottingham, Nottingham, UK; 2 School of Medicine, Academic Unit of Mental Health & Clinical Neurosciences, University of Nottingham, Nottingham, UK; 3 Tourette Syndrome Inclusion in the Community (TIC), Kingston upon Hull, UK; 4 School of Geography, Politics and Sociology, Newcastle University, Newcastle upon Tyne, UK; 5 Social Sciences Research Institute, University of Sheffield, Sheffield, UK; 6 Genius Within CIC, Plumpton, UK; 7 Neuro-diverse.org, Brussels, Belgium; 8 Tourettes Action, Farnborough, UK

**Keywords:** Developmental neurology & neurodisability, Adolescent, Neurophysiology, Quality in health care, QUALITATIVE RESEARCH

## Abstract

**Abstract:**

**Objectives:**

Chronic tic disorders (CTDs)—such as Tourette Syndrome (TS)—are neurodevelopmental disorders affecting at least 1% of the population, causing repetitive involuntary movements and vocalisations known as tics. This study aimed to explore the lived experiences of accessing healthcare for people with CTD or TS and their families in the United Kingdom (UK), as part of a larger programme of work to inform change to healthcare services for this population.

**Design:**

Informed and designed with extensive patient and public involvement, the design utilised qualitative research using focus groups. Reflexive thematic analysis was used to analyse the data.

**Setting:**

Participants were recruited via online support groups, social media and research registers.

**Participants:**

Seven focus groups were held separately with young people with tics (n=2), adults with tics (n=10) and parents/guardians of children with tics (n=11), led by a lived experience expert (coauthor PS) and facilitated by researchers. Discussion focused on three areas: the impact of living with tics, experience accessing healthcare for tics and management of tics.

**Results:**

Five themes were developed highlighting challenges across the healthcare pathway, including gaining a diagnosis, and receiving treatment, resulting in the use of self-support methods to reduce tic expression or the impact of tics. Themes also illustrated perceptions that healthcare provider's knowledge impacted initial interactions with the healthcare system, and how healthcare systems were not felt to be prioritising CTDs.

**Conclusions:**

The findings highlight a lack of prioritisation for tic disorders compounded by a healthcare structure which does not support a complex condition that requires a multidisciplinary approach. This research calls for improvements to UK healthcare services for CTD.

**STRENGTHS AND LIMITATIONS OF THIS STUDY**

- The study has benefitted from patient and public involvement (PPI) from design through to dissemination.
- PPI shaped the focus group process and questions and was led by a lived experience facilitator, improving rapport building and trust during focus groups, encouraging honest and indepth responses.
- Difficulty in finding suitable dates and times led to some initially consenting participants not following up to focus group invitations. In particular, there were a small number of participants taking part in the young people’s focus group.

## Introduction

 Chronic tic disorders (CTDs)—such as Tourette Syndrome (TS; in this article, we have used the terms ‘Chronic Tic Disorder’, ‘Tourette Syndrome’ and ‘tics’—as these are more consistent with previous research referring to healthcare practice. However, we recognise that other terms such as ‘Tourettic’ may have more meaning and relevance to the lived experience community and will use these when reflecting the lived experience voice^1 2^)—are estimated to affect at least 1% of children, young people and adults,^3^ although these figures are based on clinical samples the true prevalence is likely to be higher.^4^ Tics are repetitive, sudden, involuntary movements (motor tics) or vocalisations (vocal tics), including eye rolling, head jerking and throat clearing. Tics often emerge during early childhood—typically between 3 years and 8 years of age—often peaking in early adolescence and continuing into adulthood for many.^5^ TS is associated with increased risk of depression, anxiety and death by suicide in adulthood,^6^ social stigma and discrimination,^7 8^ reduced quality of life,^9^ poorer educational and occupational outcomes^10^ and lower economic status.^11^ Many experience secondary disability resulting from pain and injuries caused by tics^12 13^ and have co-occurring neurodevelopmental and mental health conditions which exacerbate tics.^7^ Over time, tics can worsen and become more severe and complex^14^, of which have also been associated with poorer prognosis.^1^ The impact of tics on people with CTD and their families has been well-documented^15^,^17^—with many describing significant psychological distress, poor quality of life and mental health problems affecting both their child with tics and the wider family.^7 18 19^

People with CTD need access to medical specialists who have the necessary training and expertise to assess tics and make an appropriate diagnosis. In the United Kingdom (UK)—which has a publicly-funded healthcare system—the pathway to accessing healthcare for tics requires a referral from a healthcare professional in primary care—such as a general practitioner (GP)—to a more specialised service in secondary care. However, recent evidence indicates that there are only 12 service providers in England offering a pathway for the referral, assessment, treatment and management of tic disorders in children and young people (CYP), highlighting a lack of care and regional disparity in care provision. Research has also shown delays to referral of up to 3 years on average, between tic onset and diagnosis.^15 20^ Specialist care can be accessed privately at cost; however, this also introduces further inequality in access to care.

The European and American treatment guidelines^21 22^ recommend behavioural therapy as a first-line intervention for tics and tic disorders.^23 24^ However, a lack of specialists available to provide interventions for tics was identified as one of the main barriers for young people accessing support for their CTD in the UK.^15^ Furthermore, healthcare professionals in England report needing more guidance and support with the assessment and treatment of tics in CYP, highlighting a need for workforce training and development to support healthcare for CTD.^15 20^ For many conditions, the UK’s National Institute for Care and Clinical Excellence (NICE) produces evidence-based clinical guidelines to prevent, diagnose and treat medical conditions, which can support with National Health Service (NHS) service planning and commissioning.^25^ However, these are not in place for CTD, meaning healthcare professionals in primary and specialist care do not have clear NICE guidelines for the assessment, treatment and management of tics.^12 26^ A lack of guidelines may reduce the standardisation of assessment and treatment, leaving healthcare professionals uncertain how best to operate. While the majority of research in the area has been quantitative, qualitative studies have started to explore the impact of healthcare access on service users’ lives, such as healthcare professionals’ lack of knowledge altering their treatment plans.^26^ Understanding the lived experiences of service users can provide useful insights to inform the need for guidelines and changes to services.

Building on the need to understand the lived experiences of service users, it is essential that research is clinically relevant^27^ and addresses the priorities of patients and end-users.^28^ In the early stages of developing complex interventions for healthcare, guidelines advise that research should be completed to identify and assess the problem in order to understand it.^29^ Hence, the involvement of patients and the public in developing research to ensure understanding of the topic is vital. Consequently, the aim of the current research was to explore, using qualitative methods, the experiences of children, young people and adults with tic disorders when accessing healthcare for their tics in the UK. We coproduced the research with members of the lived experience community —specifically the ‘Tourettic’ community —to ensure the aims, methods and findings were relevant to those with CTD. We also shared the findings with stakeholders involved in the design and delivery of healthcare services for CTD.

## Methods

Qualitative methods offer the potential to understand the reality for and causes of one’s experiences and are therefore are an effective way to explore the complex problems experienced by people with CTD and their family members.^30^ Focus groups were chosen for data collection to facilitate discussion and interaction between individuals with lived experience on this topic. To ensure the research met the priorities of the Tourettic community and its stakeholders, it was informed by and conducted with input from the TS steering group (TSSG) based at the University of Nottingham, which is a group of stakeholders with lived experience caring for, providing support to or those living with tics.

Given their extensive use in health research, we have followed Consolidated Criteria for Reporting Qualitative Research guidelines to increase transparency of reporting^31^ (see online supplemental additional file 1). Nonetheless, we also recognise such checklists may fail to capture the meaningfulness and the quality of qualitative research when measuring against positivist measures such as reliability and validity, and therefore the authors have also engaged in reflective practice by expanding on answers to the checklist throughout the manuscript.^32^

### Participants and recruitment

Convenience sampling was used with the aim of collecting data that gives breadth and depth to the analysis, where consideration is made to recognise where deeper understanding may be needed or new areas might require exploring during data collection, supported by taking field notes.^33^ The study was advertised on social media and through UK charities and support groups for people with tics and CTD/TS. The advertisements contained a link to the participant information sheet (hosted on JISC Online Surveys) where potential participants could learn more about the study. Parents of children and young people with tics, young people with tics aged 14–17 years and adults with tics (aged 18 and over) living in the UK were invited to participate. If interested, participants could click a link at the end of the information sheet taking them to an online consent or assent form for children. Considering the difficulties accessing diagnostic assessment in the UK, participants did not require a formal CTD diagnosis to be eligible to participate but confirmed they were on a waiting list for assessment if no diagnosis was in place or had prior experience of seeking a referral to specialist services for tics.

### Focus group procedure and data collection

Once consent was obtained, the online survey automatically directed participants (or their parents) to a form designed to collect demographic details. Young people with tics (aged 14–17 years) were given the option for their parent/caregiver to also join their focus group.

All participating individuals were sent a familiarisation video prior to the scheduled focus group. These brief videos (<2 min) consisted of the focus group facilitators introducing themselves, their role in the research study and describing the types of questions that would be asked during the focus groups. Participants were also sent a ‘code of conduct’ outlining some basic principles for taking part in the focus groups such as confidentiality and respect for others’ views and experiences.

Participants were sent a link to an online focus group (held and video recorded by Microsoft Teams) and advised that the discussion could last up to 90 min. Members of the research team (JS and CMB) and one lived experience expert (PS) were present in all focus groups, with PS being the focus group facilitator directing the questions. The facilitator’s quotes were included in the analysis as a lived experience expert. Focus groups followed a semistructured format, beginning with an ice-breaker question to help participants feel at ease, followed by a reminder of the code of conduct for participation. The questions presented to each of the groups of participants were the same, with small changes made to the wording to reflect the participant group:

1. How has your journey been when accessing support for your tics/your child’s tics?
2. Do you think your/your child’s life would have been different if your medical journey was different?
3. What are your experiences of living with/caring for a child with TS or with a tic disorder?
4. What do you do/your child do to help with your symptoms?

Each focus group finished with a debrief which included contact details of support groups. A £15 voucher was given to participants as a gesture of thanks for their time. The topic guides for the focus groups are presented in online supplemental additional file 2.

### Data analysis

The research team followed a process for inductive reflexive thematic analysis,^34^,^36^ aiming to mirror the meaning communicated by participants.^37^ Data were transcribed using an automated transcription service and checked by JS for accuracy, and identifying details were redacted. Participants were not asked to review the transcribed data. Following a period of familiarisation of the data, initial codes pertinent to the research questions were generated by JS within each focus group transcript. Initial codes and a coding system were stored on MS Excel spreadsheet software to aid offline and online collaboration between members of the research team (CMB, MG, JS). Through an iterative review process, the initial codes were collected and placed into increasingly broader subcategories and eventually derived into themes. These were extensively discussed and reviewed by the researchers to ensure the data were treated with cohesion and consistency. These discussions included reflections on what was felt to be important to the PPI team members, including whether to analyse and report data from each participant group separately or together. Codes from each participant group were initially developed and held separately, but given the overlapping areas, codes from each group were brought together but indexed and colour-coded to reflect their participant group, allowing researchers to keep note of whether certain initial themes, themes or subthemes were group-specific. Such instances have been included in the findings. The final themes were checked with the wider research team, and PPI members were invited to make changes to the themes. A reflexive statement is offered by JS, CMB and PS in online supplemental additional file 3, and reflexive field notes were made by the research team.

### Patient and public involvement statement

Adopting the slogan ‘nothing about us, without us’, incorporating the lived experiences of those accessing healthcare into practice is gaining recognition as a valuable approach.^38^ The rationale and design of this research study were informed by four PPI members, all co-authors on this paper, and input was also given by the TSSG. The PPI members of the team included a parent of a young person with TS (EM) who also leads a national UK charity (Tourettes Action), an adult with TS who also works for a community group supporting those with tic disorders (PS), an adult with TS who is also a lived experience researcher (DPJ) and a research psychologist (SA) who does not have lived experience of TS, but has a professional background in supporting involvement in research of those with lived experience of neurodiversity, including TS.

These team members informed the study methods including participant recruitment strategies, information sheets and consent forms, the focus group questions and the conduct and setting up of the focus groups and the debrief sheet. They reviewed and amended terminology in all documents, ensuring these would be appropriate for the lived experience community. PS facilitated all focus groups alongside the research team. All PPI members helped interpret the themes developed from the analysis and co-authored the paper. They were instrumental in disseminating the study findings via a short animation promoted on social media through their networks^39^ . For an overview of how PPI shaped the research, see online supplemental additional file 4 for the Guidance for Reporting Involvement of Patients and the Public 2 (GRIPP2) checklist.^27 40^

## Results

### Results

In total, 23 participants took part across seven focus groups between May and July 2022 (table 1). Separate focus groups were held for each group of participants, and each group had participants who were lost to follow-up after consenting and becoming uncontactable or unable to attend focus groups.

**Table 1:** A summary of the participants included in each focus group

Adults with tics			Young people with tics	Parents/guardians of a child with tics		
Focus group 1	Focus group 2	Focus group 3	Focus group 1	Focus group 1	Focus group 2	Focus group 3
AF1	AF1	AF1	AF1	AF1	AF1	AF1
A1	A6	A8	YP1	P1	P5	P7
A2	A7	A9	YP2	P2	P6	P8
A3		A10	YP2-P	P3		P9
A4				P4		P10
A5						

A, adult with tics; AF1, adult facilitator with tics (PS); P, parent/guardian of a child with tics; YP, young person with tics; YP2-P, parent of a young person with tics.

### Adults with tics

30 adults with tics (aged 18+ years) registered their interest to participate and consented to take part. Three focus groups were completed with 10 adults with tics (median age 34 years, range 18–57, 4 men, 4 women, 1 non-binary participant, 1 transgender participant) lasting between 1 hour 25 min and 1 hour 45 min.

### Parents/carers of people with tics

41 parents of children aged 17 years or under responded to the recruitment call and 39 parents gave consent for themselves to take part. Three focus groups were completed with 11 participants in total (median age 45 years, range 35–55, 11 women), with between two and five participants in each, lasting between 1 hour 24 mins and 1 hour 33 mins.

### Young people with tics

19 young people with tics (aged 14–17 years) registered interest, and 17 young people assented to take part. Of these, seven young people’s parents also gave consent for them to take part. One focus group was held for young people, consisting of two young people (n=2, median age 15 years, range 16–17, 1 male) and a parent of a young person, lasting 1 hour 13 min.

### Findings

Five themes were derived from seven focus groups with 23 people with lived experience of tics—either from experiencing tics themselves or as a parent of a child/young person with tics. We provide an overview of the themes (table 2), which have been ordered in relation to the stages of the healthcare journey discussed in the focus groups.

**Table 2:** Themes derived from reflexive thematic analysis

Theme 1	Lived experiences of interactions with healthcare professionals feel more positive when the professional has an understanding of TS.
Theme 2	Getting a diagnosis acts as a gatekeeper to information, support and acceptance, a lack of which can delay or prolong difficulties.
Theme 3	A lack of available treatment, limited treatment options and fragmented access to care causes barriers when accessing treatment.
Theme 4	Preventative strategies are used to support and manage tics at home.
Subtheme 4a	Socialising leads to spaces for support, sharing and learning online and in-person.
Theme 5	The healthcare system does not feel to be structured in a way that prioritises and supports TS.

TS, Tourette Syndrome.

At the first stage of a healthcare journey, healthcare professionals may be approached for support or diagnostic assessment. Participants described a range of experiences resulting from this initial interaction with healthcare providers that were influenced by the professional’s knowledge of the condition (Theme 1). For participants who were able to obtain one, a diagnosis was reported as a gateway to access more support and information about tics and can improve self-acceptance (Theme 2). However, irrespective of diagnosis, the analysis highlighted poor access to treatment and limited treatment options (Theme 3). In response to poor access to treatment, participants described using different methods to prevent a deterioration in their symptoms or for self-care (Theme 4), including the importance of socialising (Subtheme 4a). Overall, Theme 5 brought together views that TS is not prioritised and that neurodevelopmental disorders do not fit within the structure of current UK healthcare systems.

#### Theme 1: “You're only as good as who you're seeing” – lived experiences of interactions with healthcare professionals feel more positive when the professional has an understanding of TS

Different experiences with medical professionals were heavily influenced by the professional's understanding and prior experience of tics and CTD. For example, interactions with professionals who were supportive and knowledgeable led to positive experiences that facilitated care and treatment for the individuals: “*I was quite lucky with my GP…. The GP knew a lot about Tourettes”* (A10). Whereas those who saw healthcare professionals who did not understand tics or TS, left participants feeling neglected: “*they don’t understand the issue at hand and that’s what the frustration is”* (P8).

Healthcare professionals who had experience and understanding of the condition appeared to put in extra time and dedication to working with patients and their families: *“one particular doctor, who made it her life goal to make sure that my child got some help. Every time he got knocked back, she’s like ‘well, I’m not putting up with this”* (P2). In contrast, professionals viewed to be less supportive were dismissive or appeared disengaged: *“[I] actually had to ask them at my next appointment a few months later, 'is this Tourette’s?' Because I’d googled it and that was the only thing that came up under that name”* (A7).

Specifically, the adult group highlighted the need to receive more information about their condition from a healthcare provider as a means to provide psychoeducation but also to help reduce stigma: “*if I'd have come away with that, had that info that was given to me, not just Google by myself or you know like kind of scrabbling around trying to find information about something… it’s just if you’d have had that [information] then I think it would have eliminated a lot of the shame really”* (A1). Adults suggested signposting would be useful to understand CTD: *“when I did get diagnosed and I never heard back from anybody, they never asked me to, they never sent me anywhere, they never gave me any links or any, you know, groups to join or anything like that”* (A5).

#### Theme 2: “That day when you told me what it was, changed, literally changed my life because suddenly, [I had] an explanation for things” – getting a diagnosis acts as a gatekeeper to information, support and acceptance, a lack of which can delay or prolong difficulties

A diagnosis was a catalyst in getting the right support for people with CTD, both within and outside healthcare settings. In their personal lives, participants highlighted how being able to attribute their behaviours to CTD provided explanations, for example, with job interviews or challenging behaviour in school: “*[I] possibly could have been diagnosed with… ADHD and Tourette’s then, but I was just, you know… a naughty child, and that really really upset me”* (A5). Parents felt that they might have parented differently had they known their child had a diagnosis earlier: *“it created a lot of anxiety and problems at home… we were, you know, making her go to bed at ridiculous times”* (P3).

Within the healthcare system, access to the right treatment without a diagnosis became very difficult or delayed: *“What we would have avoided if we’d have had a correct diagnosis, we would have avoided a lot of the things like referral to dieticians”* (P3). Sometimes, it was felt that treatment came too late: *“earlier help probably would have helped a little bit, especially with school, because there have been times where my tics have been so bad that I haven't been able to sit inside of a classroom, which meant that times I’ve lost parts of my education”* (YP2). In addition, the act of receiving a diagnosis leads to an acceptance process: *“the fact that you know that I’d, I’d kind of reached out to this person to say 'look, I’m now ready to engage and ready to kind of admit that there’s something wrong that I’ve been kind of trying to hide'”* (A1). This can be important for the person with CTD to go through in order to understand their condition better: “*[the diagnosis] helped me understand what it was better, so I initially, there was a bit of almost grieving 'cause I was like, I’ve kind of hoped that they were just going to say 'oh well, it’s just a habit and it will go away, you’ll be fine, you’ll get on with it, be good in 10 years', but it did, initially it hit me hard and I felt really bad about it for a while”* (A6).

Nonetheless, while receiving a diagnosis may act as a gateway to better support and understanding, obtaining a diagnosis was rarely an easy process: “*I said to her, look, I’m having these real, real issues here and she said, and I quote verbatim, ‘the NHS does not have a mechanism to diagnose Tourette’s in adulthood’. Puts the phone down”* (A1), and, “*I said 'for the love of God, just refer me. You don’t even have to confirm it yourself, just refer me and get the neurologist to just check me out'”* (A8). Several participants reported the need to turn to private healthcare for a diagnosis “*because it [the NHS] was just such a long process”* (A5).

#### Theme 3: ‘‘I’m just finding it very difficult to actually access any of that [treatment]’ – a lack of available treatment, limited treatment options and fragmented access to care causes barriers when accessing treatment

In terms of getting treatment for tics, it was regularly reported that treatment can be difficult to access: *“I’ve gone to the doctor and said 'I’ve just got this tic and it’s non-stop and I’m getting injuries from it' and they’re just like ‘oh, what do you expect us to do?'”* (A6).

The parent group highlighted that access to treatment is usually only available via an out-of-area referral, “*I have never ever seen a Tourette specialist. I go for support to the local, I’m saying locally, it’s a 125-mile round trip away”* (AI1), and “*the only people that will treat me are in London, so it’s a long way away*” (A4). This has led to people referring to access to specialist care as “*a bit of a postcode lottery”* (P4).

Furthermore, a view held by mostly parents included the eligibility criterion for specialist support being restrictive, meaning certain criteria (e.g., severity, age, symptoms) must be met for successful referral. For example, some parents highlighted that only the most urgent cases are seen: “*the only time you get any reaction from CAMHS [Child and Adolescent Mental Health Services] is if you’re showing some symptoms of harm”* (P9) and “*there was no way on the planet they were gonna send him [to a specialist TS centre]”* (P6).

For those receiving behavioural treatment, problems included difficulties developing rapport with the therapist: *“he was really patronising”,* or any sense of personalising treatment to the young person: “*they made no adaptation whatsoever”* (P5) and without sustained support “*you get six sessions and then that’s it. Bye bye. You’re discharged”* (A10]. Similarly, for those receiving medications, which several mentioned as being frequently suggested by healthcare professionals, the type of medication was perceived as inappropriate: *“he should never have been given those medications, especially the antipsychotics”* (P6). Such difficulties can add barriers to engaging with support.

#### Theme 4: “All of my techniques have all been my own suggestion, I’ve got various different coping strategies, and nobody’s ever suggested them except for me” – preventative strategies are used to support and manage tics at home

Many participants reported using self-made methods to manage their tics. These often centred on using calming spaces that might reduce the intensity of one’s tics: “*making it a nice calm household”* (P1). This included having pets, “*I’ve got birds and I love them… ‘cause they’re so tiny I just hold them and it makes my tics just shut up*’ (A4) and being out in nature, “*it’s always nice to sort of get out and I go do photography and do long walks and I find that the walks actually limit the tics because it brings my body and mind into line energy-wise*” (A10). In addition, for some participants, distraction was noted to lighten tics, with family members coming up with the suggestions: “*…quick distract her and say something about one of her favourite shows or tell her a little bit of gossip*” (P1) and “*I really like painting and playing games and it’s just a nice little step back when I need to have a little bit of a breath to calm me down”* (YP2).

Participants also created their own methods to prevent harm or injury that can arise from tics, for example, using joint protections, “*she wears sort of like knee supports”* (P9) and “*I wear compression braces on my knees”* (AF1)—which were also suggested within family and friend circles. Finally, items such as fidget toys redirected or altered tic expression: “*fidget toys, because they help keep my hands busy… So then, instead of destroying my hand, I destroy a stress ball instead”* (A7).

#### Subtheme 4a: “It’s groups like this, where you learn from each other. That’s how we get through day by day, not from the NHS” – socialising leads to spaces for support, sharing and learning online and in-person

Participants felt the support they gained via in-person or virtual social groups was more useful than from healthcare providers: *“I seem to have to find more information from the people around me [friends with Tourette’s and people that have tics] than I can from like looking up or talking to professionals”* (A4) and *“we started talking [on Instagram] and now we’ve met… without them, I would not be anywhere near as confident with my Tourette’s as I am now”* (A2). One of the commonly reported benefits of socialising was to share experiences with people who had been through similar situations: “*discussing how it affects your life, and then it’s the psychological support”* (A1).

#### Theme 5: “I would say it needs to be like a combined service like, it, it probably isn’t just 'one or the other'” – the healthcare system does not feel to be structured in a way that prioritises and supports TS

CTDs are complex conditions, meaning that someone might need support from multiple professionals within the healthcare system resulting from their CTD. Participants highlighted support needs beyond tic symptomology (e.g., reducing tic frequency), for example, on their mental health: *“Actually, it’s not necessarily the tics that need support, it’s actually the after-effect, it’s the, you know, it’s the parallel effect it has as well on your well-being”* (A1). Sometimes, support is needed for pain, *“how to kind of eradicate some of the pain”* and other occasions for tic frequency, *“it would also be great to know how I can help reduce my tics”* (A1).

Most participants highlighted that the healthcare system does not function to support complex needs, with many parents describing a vicious cycle of not receiving support, “*we were back and forward to accident and emergency all the time, and obviously they referred us to Child and Adolescent Mental Health Services because it was mental health, and then they said she was too complex so couldn’t actually see her anyway”* (P10). Participants also described a mismatch in the delivery of behavioural therapy and complexity of tics, for example, not seeing it as suitable for people with multiple tics in different body regions, *“if I was getting repetitive tics in my shoulders, my arms, my legs, I need help with all of those pieces, but they’d say, right? Let’s focus on one”* (A6).

Finally, participants felt that CTDs are not perceived as a high-enough priority to address within the healthcare system and described a sense that other conditions receive more attention, *“children like her don’t get a look in”* (YP-P1) and a feeling that other conditions would be prioritised: “*it’s almost like this lack of support just impacts all facets of the child, of the, you know, the Tourette’s sufferers, a tic sufferer’s life. It’s just, it’s just it’s cruelty… however, they dress it up - not enough budget, not enough understanding. I don’t care, I just, I quite frankly don’t care because if he had a broken leg, it’ll be fixed. That’d be fixed*” (P8).

## Discussion

The core aim of this project, codeveloped with the CTD community, was to capture and share the experiences of accessing healthcare for tics in the UK. Themes highlighted challenges across the healthcare pathway, from gaining a diagnosis to receiving treatment and highlighted the use of self-made support methods to reduce tic frequency, onset or to protect oneself from tics. Finally, themes captured the sense of a lack of prioritisation for CTD by healthcare providers. Using qualitative methods, we were able to explore healthcare experiences while integrating the priorities of those with lived experience for the research questions and dissemination, a design method that can be useful for sparking ideas for support.^1 30^

Themes support previously published research indicating a lack of specialist care for CTD^15 41^ and provide valuable insights into the impact of this, with participants highlighting the difficulties obtaining a diagnosis (Theme 2) and others travelling long distances to secure treatment. The value of peer support services/networks was also identified (Theme 4a). The results further suggest that only the most urgent cases are receiving support, with little access for those who may be in need but not at crisis point (Theme 4) or only for those who are willing to pay privately for healthcare (Theme 2). These qualitative reports are in accord with quantitative findings from a UK-based survey where only just over half the sample were immediately referred to specialist secondary care and 30% of respondents highlighted significant difficulties in accessing care.^42^ Findings from a recent mixed-methods study have identified as little as 12 care boards within the UK that have a clinical service pathway for assessment and treatment of tics, with the majority of these found within London.^20^ The delayed diagnoses—as mentioned by several participants in the current study—indicate many problems within the healthcare system (such as non-existent clinical pathways)^43^ leading people to access healthcare privately, as also noted by Marino *et al*.^42^ Altogether, these findings indicate that healthcare for CTD does not align with the NHS’s fundamental aim to provide universal healthcare access, and the NHS ‘Long Term Plan’ to reduce health inequalities.^44^ Ensuring the development of healthcare policies that support improved design and implementation of healthcare services for CTD across the UK is necessary. Without improvements, healthcare services are likely to continue to see an increase in waitlists and workloads, as already evident in other neurodevelopmental conditions.^45^ By prioritising the needs of people with CTD, the NHS can support equal access to care, reducing health disparities between those who can and cannot afford to travel further afield or fund private healthcare.

In terms of what healthcare for CTD might need to look like, the themes presented in this study revealed that current healthcare provision does not account for the variety of symptoms experienced by someone with CTD. Care was revealed to be lacking at points of early access in the scarce information provided to the patient (Theme 1) and in the treatment being delivered not accounting for both the individual attending therapy or the interplay between differing tics and the emergence of other co-occurring conditions over time (Theme 3). This supports observations from a meta-synthesis of qualitative studies which concluded ‘*services require multifaceted approaches to support individuals in a comprehensive manner, where educational, social and vocational factors are equally considered*’ (p.629,^46^). Without coordinated care, accompanying conditions may go undiagnosed and untreated, which can lead to greater dysfunction than by tics alone and can confound treatment plans.^47^ Ideally, holistic care would not be isolated to healthcare settings and would involve support at home, school and work, where people with TS face further challenges. For example, psychoeducation and socialisation, identified in this study to be helpful management techniques, could be employed under such care. To counter challenges relating to living with multiple conditions where treatment plans may need to change, personalised care could ensure that treatment is tailored to the needs of the patient. A multidisciplinary and personalised care model—encompassing healthcare professionals across different medical specialisms and sectors—could overcome the siloed infrastructure of the NHS^48^ and focus on functional impairment rather than medical management, acting as preventative support for the person living with tics across the life course.^47^ Finally, as established tic services are geographically restricted to southern parts of the UK,^20^ another potential option within tic pathways is to use digital options to assist healthcare access and treatment, such as videoconferencing or telehealth, to support consultations between patients and practitioners and provide behavioural therapy online.^49^ Freedom of information requests suggest such methods are not currently used routinely in the UK for tic disorders.^20^

A further implication highlighted by the findings relates to the need for further training of medical professionals on CTD. One of the themes centred on the difference between interactions with professionals who had greater understanding of CTD or who showed a willingness to learn, compared with those who did not. The former led to participants feeling supported and understood, whereas the latter left participants feeling neglected and ashamed (Theme 1). Likewise, professionals with little knowledge while delivering treatment can be ineffective if rapport is not developed or incorrect medications are given (Theme 3). These findings corroborate previous publications: one survey reported 10% of GPs had limited knowledge of tics,^50^ and a more recent survey highlighted only 14% of GPs referred to tics or a CTD during their first consultation.^42^ Additionally, in a worldwide survey of movement disorder clinicians, only a quarter (27%) felt confident in the knowledge of CTD pathophysiology.^51^ Finally, a survey of healthcare professionals in England identified a need for more training and support in assessing and treating tics in CYP.^20^ Together, these findings raise striking limitations in the training in CTD given to healthcare professionals. Nevertheless, the themes also stress the importance of care and understanding by healthcare professionals when patients are seeking support. Given the role of stigma as a help-seeking barrier for TS, there is a need for healthcare professionals to be empathetic and compassionate;^15^ such care has been linked to improved outcomes for patients.^52^ These skills can be taught; a patient-led programme to improve empathy and knowledge on TS and associated conditions led to improved physician empathy and empowered patients.^53^ Training on CTD should include a focus on building empathy and compassion as well as increasing knowledge of tics. Building this into the healthcare curriculum could result in improved well-being for both patients and healthcare workers, but also lead to patients feeling better understood, with improved care outcomes and reduced stigmatisation.

Finally, in discussing these various findings and their implications, something not to be overlooked here is that all these individuals—patients, their families and healthcare professionals—do not exist in a vacuum. Multiple environmental forces—from the micro (i.e., individual) to macro levels— influence how we conceptualise health and illness, as well as how social and healthcare systems, typically guided by national policy, respond to patients. In applying the socioecological model to tic disorders, Pring *et al*^54^ describe how the model’s different levels lead to various kinds of stigmatisation of tic disorders. At a societal/structural level, tics are commonly misunderstood and judged negatively, leading to discrimination and stigmatisation—while at a community level within the UK, there is variation in how knowledgeable healthcare professionals will be about tic disorders and the potential here for misinformation to lead to stigmatisation of patients. In approaching the issue of healthcare inequalities and access in tic disorders, this paper highlights that a multi-disciplinary approach encompassing medicine, psychology, sociology, politics and more, must be taken into account moving forward.

### Strengths and limitations

A major strength of this work lies in the coproduction with partners ranging from those with lived experience of tics or caring for people with tics, to tic charity and support group representatives. Lived experience expertise contributed to all stages of the project, including the project initiation around research aims and questions to be asked, to the design, data collection, interpretation of themes and preparing the manuscript for publication. Having a lived experience facilitator enhanced rapport building and trust during the focus groups, which enabled participants to speak honestly about their experiences, promoting in-depth responses and supporting a sharing dynamic within the group. This affirms recently published work by Jones and Phoenix-Kane (2025)^55^ that explains how shared experiences between Tourettic researchers and participants influence trust and comfort of research participants.

Limitations of the project include the low response rate of young people to attend the focus groups. With only two young participants (aged under 18 years), parents became the ‘voice’ for both young people and children, meaning their experiences might not reflect what would have been heard directly from young people. The entire sample of parents was female, meaning the perspective of fathers is missing in the data. This is common when collating caregiver experiences for TS and therefore different methods may need to be explored to gather data from other caregivers. While this may also highlight societal expectations of who is responsible for childcare and the expected gender norms in society,^56^ the experiences of fathers need further exploration. In addition, a technical problem with the survey prevented the recording of ethnicity, diagnoses and co-occurring conditions, meaning we are unable to comment fully on the demographics of those involved. Finally, the challenges of finding dates and times to suit a large number of participants plus a narrow time-frame meant that several of those who initially consented to take part were not included in the final sample. This may have led us to miss the views and experiences of those from the initial consenting sample.

## Conclusion

Using a qualitative approach and participatory research methods, we collaborated with members of the tic community to understand the impact and challenges caused by the current lack of access to UK healthcare services for those with TDs. Findings from across twelve focus groups with adults and young people with tics and parents of young people with tics, using a reflexive thematic analysis, highlighted aspects from across the healthcare journey highlighting the impact of inadequate support for people living with or supporting those with tics. Taken together, the themes illustrate feelings including a lack of prioritisation for CTD within the NHS and a structure that does not support a complex condition requiring a multidisciplinary approach. An implementation of policy surrounding treatment guidelines could endeavour to create structure within the healthcare pathways, so that patients with tics have clearer routes to care and healthcare professionals are better able to direct care and could overcome these challenges. Furthermore, clinical guidelines are essential to underpin access to care for all those seeking support for tics.

## Supplementary material

10.1136/bmjopen-2024-098306 online supplemental file 1

10.1136/bmjopen-2024-098306 online supplemental file 2

10.1136/bmjopen-2024-098306 online supplemental file 3

10.1136/bmjopen-2024-098306 online supplemental file 4

## Data Availability

Due to the sensitive nature of this topic, the datasets generated during the current study are available from the corresponding author upon request due to their containing sensitive and personal information. Further information can be found at the University of Nottingham data repository (doi: 10.17639/nott.7597).
