# Supplementary material for: ‘For the love of God, just refer me’: a co-produced qualitative study of the experiences of people with Tourette Syndrome and tic disorders accessing healthcare services in the UK
Source: BMJ Open. 2025 Sep 5;15(9):e098306. doi: 10.1136/bmjopen-2024-098306 (PMC12414177; doi:10.1136/bmjopen-2024-098306)
Supplement: online supplemental file 2 [file bmjopen-15-9-s002.docx]

# Supplemental File 2: Topic guides for the focus groups with three different population groups

**1. Topic guide for focus groups with children and young people with tics/tic disorder**

| **Facilitator** | **Timings** | **Question** | **Prompts/other** |
| --- | --- | --- | --- |
| Jen | 00:00-00:15 | ***PRESS RECORD ON TEAMS TO START RECORDING THE MEETING.***  Introduce the project: The aim of this project is to raise awareness of some of the realities of living with tics and accessing healthcare   - 1. This meeting is being recorded and transcribed automatically via Teams   2. Cover confidentiality and anonymity   3. Ask them to be open and honest in their responses   4. Explain you can withdraw at any time   5. Explain you are not a medical professional   Introductions   - Introduce the research team - Introduce Woven Ink   - If you’re happy for your voice recordings to potentially be used in the campaign video, please set this up now.  1. Explain process    1. Please keep your mics muted when you’re not talking. We will mute you if you forget to minimise background noise.    2. Please use the raise hand function if you have something to say. We want to hear from everyone, but no one will be forced to talk if they feel uncomfortable    3. 5 minute break just before the hour    4. Any tech problems, please talk to Jen   Please remember that Woven Ink will be using what you have said to create videos that capture your experiences of accessing healthcare for your tics. When you answer to these questions, I may try and pry a bit more about your example and this is why you might hear a lot of follow up questions! | Introduction *– explain you are not a trained medical professional and so will not be able to provide advice.* |
| Jen | 00:15-00:35 | Icebreaker - **If there was one thing you wanted someone to know about having tics, what would it be?** | Specific group of people? Friends, people at school, peers, the public, family |
| Paul | 00:35-00:55 | **1. How has your journey been accessing support for your tics?** | How **easy** or **difficult** has it been?  How could it have been easier for you?  How long it took to diagnose, how you were treated by medical professionals?  What has been the most difficult aspect of your medical journey?  Have there been times when you’ve needed more or less support? |
| Paul |  | **2. Do you think your life would have been different if your medical journey was different?** | How long it took to diagnose?  Treatment options?  Do you still feel there is something lacking in your medical care? |
|  | 00:55-01:00 | 5 MINUTE BREAK | |
| Paul | 01:00-01:15 | **3. What are your experiences of living with Tourette’s Syndrome or with a tic disorder? We want to hear about positive and negative things** | I can hear that it hasn’t been easy, but on reflection have there been any positive experiences that you can think of.  What are your experiences of stigma (*negative or not so nice attitudes and comments from others*) related to your tics?  How has this changed from since you were diagnosed/ started ticking? |
| Paul | 01:15-01:25 | **4. What do you do to help with your symptoms?** | Exercise, relaxing, meditation, walking, music, medication, talking to others with tics etc. |
|  |  | Is there anything we haven’t talked about today that you would like to say? |  |
| Jen | 01:25-01:30 | Final comments/remarks/thank you’s | Thank participant for their time  Debrief (and send a debrief after)  Explain how they’ll get voucher sent to them |

**2. Topic guide for focus groups with adults with tics/tic disorder**

| **Facilitator** | **Timings** | **Question** | **Prompts/other** |
| --- | --- | --- | --- |
| Jen | 00:00-00:15 | ***PRESS RECORD ON TEAMS TO START RECORDING THE MEETING.***  Introduce the project: The aim of this project is to raise awareness of some of the realities of living with tics and accessing healthcare   - 1. This meeting is being recorded and transcribed automatically via Teams   2. Cover confidentiality and anonymity   3. Ask them to be open and honest in their responses   4. Explain you can withdraw at any time   5. Explain you are not a medical professional   Introductions   - Introduce the research team - Introduce Woven Ink   - If you’re happy for your voice recordings to potentially be used in the campaign video, please set this up now.  1. Explain process    1. Please keep your mics muted when you’re not talking. We will mute you if you forget to minimise background noise.    2. Please use the raise hand function if you have something to say. We want to hear from everyone, but no one will be forced to talk if they feel uncomfortable    3. 5 minute break just before the hour    4. Any tech problems, please talk to Jen   Please remember that Woven Ink will be using what you have said to create videos that capture your experiences of accessing healthcare for your tics. When you answer to these questions, I may try and pry a bit more about your example and this is why you might hear a lot of follow up questions! | Introduction *– explain you are not a trained medical professional and so will not be able to provide advice.* |
| Jen | 00:15-00:35 | Icebreaker - **If there was one thing you wanted someone to know about having tics, what would it be?** | Specific group of people? Friends, the public, family, colleagues |
| Paul | 00:35-00:55 | 1. **How has your journey been accessing support for your tics?** | How long it took to diagnose, how you were treated by medical professionals?  What has been the most problematic aspect of your medical journey?  Have there been times when you’ve needed more or less support? |
| Paul |  | 1. **Do you think your life would have been different if your medical journey was different?** | Length of time to diagnosis, do you still feel there is something lacking in your medical care |
|  | 00:55-01:00 | 5 MINUTE BREAK | |
| Paul | 01:00-01:15 | 1. **What are your experiences of living with Tourette’s Syndrome or with a tic disorder? We want to hear about positive and negative things** | What are your experiences of stigma related to your tics?  How has this changed from since you were diagnosed/ started ticking? |
| Paul | 01:15-01:25 | 1. **What do you do to help with your symptoms?** | Exercise, relaxing, meditation, walking, music, medication, talking to others with tics etc. |
|  |  | Is there anything we haven’t talked about today that you would like to say? |  |
| Jen | 01:25-01:30 | Final comments/remarks/thank you’s | Thank participant for their time  Debrief (and send a debrief after)  Explain how they’ll get voucher sent to them |

**3. Topic guide for focus groups with parents of child/young person with tics/tic disorder**

| **Facilitator** | **Approx. timings** | **Question/content to be covered** | **Prompts/other** |
| --- | --- | --- | --- |
| Jen | 00:00-00:15 | ***PRESS RECORD ON TEAMS TO START RECORDING THE MEETING.***   1. Introduce the project   The aim of this project is to raise awareness of some of the realities of living with tics (including within wider family) and accessing healthcare   - 1. This meeting is being recorded and transcribed automatically via Teams   2. Cover confidentiality and anonymity   3. Ask them to be open and honest in their responses   4. Explain you can withdraw at any time   5. Explain you are not a medical professional   Introductions   - Introduce the research team - Introduce Woven Ink   - If you’re happy for your voice recordings to potentially be used in the campaign video, please set this up now.  1. Explain process    1. Please keep your mics muted when you’re not talking. We will mute you if you forget to minimise background noise.    2. Please use the raise hand function if you have something to say. We want to hear from everyone, but no one will be forced to talk if they feel uncomfortable    3. 5 minute break just before the hour    4. Any tech problems, please talk to Jen   Please remember that Woven Ink will be using what you have said to create videos that capture your experiences of accessing healthcare for your child’s tics. When you answer to these questions, I may try and pry a bit more about your example and this is why you might hear a lot of follow up questions! | Introduction *– explain you are not a trained medical professional and so will not be able to provide advice.* |
| Jen | 00:15-00:35 | Icebreaker - **If there was one thing you wanted someone to know about having tics, what would it be?** | Specific group of people? Friends, the public, family, colleagues  What it’s like as a family?  Do you feel these [issues] are understood by the individuals who your child interacts with? |
| Paul | 00:35-00:45 | 1. **How has your journey been accessing support for your child’s tics?** | How long it took to diagnose, how you and your child were treated by medical professionals?  What has been the most problematic aspect of your medical journey?  Have there been times when you’ve needed more or less support, either for your child or for yourself?  What additional support would be most appreciated? It’s been noticed that often parents and carers use words like ‘fight’ or ‘warrior’ to describe their role in getting the help that their child needs. Could you comment on that? |
| Paul | 00:45-00:55 | 1. **Do you think your child’s life would have been different if your medical journey was different?** | What are you hoping for their medical journey in the future?  Have you found medical professionals empathetic?  Have there been any medical professionals who were particularly helpful?  Length of time to diagnosis, do you still feel there is something lacking in your child’s medical care? |
|  | 00:55-01:00 | 5 MINUTE BREAK | |
| Paul | 01:00-01:15 | 1. **What are your experiences of caring for a child living with Tourette’s Syndrome or with a tic disorder? We want to hear about positive and negative things** | For parents especially  What are your experiences of stigma related to being parent/caring for child with tics?  How has this changed from since your child was diagnosed / started ticking? |
| Paul | 01:15-01:25 | 1. **What sort of things do you and your family do to help your child cope and manage their symptoms?** | Sibling involvement, grandparents, family activities or giving the child more space and time to decompress?  Have you reached out or tried to find other families going through similar issues? Where was this? Good and not-so-good aspects of this? |
|  |  | Is there anything we haven’t talked about today that you would like to say? |  |
| Jen | 01:25-01:30 | Final comments/remarks/thank you’s to participants | Thank participant for their time  Debrief (and send a debrief after)  Explain how they’ll get voucher sent to them |
