# Supplementary material for: ‘For the love of God, just refer me’: a co-produced qualitative study of the experiences of people with Tourette Syndrome and tic disorders accessing healthcare services in the UK
Source: BMJ Open. 2025 Sep 5;15(9):e098306. doi: 10.1136/bmjopen-2024-098306 (PMC12414177; doi:10.1136/bmjopen-2024-098306)
Supplement: online supplemental file 3 [file bmjopen-15-9-s003.docx]

## **Supplemental File 3: Author reflexivity statements**

## ***Reflexivity statements for the three authors directly involved in running the focus groups and analysing/interpreting the data (Babbage, Salvage, Stevenson) are provided:***

**CB Reflexive Statement:** CB is an experienced qualitative and lived experience research fellow, and has had involvement across many projects carrying out data collection and analysis utilising reflexive thematic analysis. They completed a PhD looking into the development of wellbeing tools for young people with TS, and since starting the doctoral training has developed and maintained a strong relationship with the tic community, including volunteering at events and keeping in touch with members. Therefore, CB has an awareness of the impact of low access to healthcare for those living with TS. Furthermore, having relatives who are neurodiverse means CB has their own lived experience by proxy of the impacts and challenges that can come from living with neurodevelopmental disorders. CB is a white British, heterosexual, cisgendered female.

**JS Reflexive Statement:** JS has completed a PhD investigating plasticity differences in those diagnosed with TS, using non-invasive brain stimulation techniques. During this extended period of study JS connected with young people and adults with TS, and their families, often spending multiple days with them during the research. On many occasions the community would want to engage in discussion about their ability to access support, or the difficulties they had experienced with healthcare. These extensive shared communications, having their own diagnosed neurodiversity, and having personal experience of struggling for a diagnosis and necessary medical support, means JS has a great deal of empathy for those in the tic community. JS is a white British, heterosexual, cisgendered female.

**PS Reflexive Statement**: PS is a Lived Experience Ambassador with Genius Within and an advocate for the Tourette’s Syndrome and neurodivergent communities. Having been diagnosed later in life with Tourette’s Syndrome, ADHD, OCD, and sensory processing disorder, PS has a profound understanding of the challenges faced by those living with neurodevelopmental conditions. His advocacy work includes contributing to organisations such as Tourettes Action, Tourettes Scotland, and Tic & Hull Yorkshire, as well as supporting international efforts in countries like Italy. Through his lived experiences of navigating educational, employment, and healthcare systems, PS is deeply aware of the barriers to accessing appropriate support. Being listed in the Shaw Trust Disability Power 100 for 2024 reflects his dedication to amplifying voices within the neurodivergent community. Paul is a white British, heterosexual, cisgendered male.
