# Supplementary material for: ‘For the love of God, just refer me’: a co-produced qualitative study of the experiences of people with Tourette Syndrome and tic disorders accessing healthcare services in the UK
Source: BMJ Open. 2025 Sep 5;15(9):e098306. doi: 10.1136/bmjopen-2024-098306 (PMC12414177; doi:10.1136/bmjopen-2024-098306)
Supplement: online supplemental file 4 [file bmjopen-15-9-s004.docx]

## **Supplemental File 4: GRIPP2 Checklist**

| 1: Aim | This study aimed to incorporate the lived experiences of individuals affected by Tourette Syndrome (TS) into the research process. Given the rationale of this research was informed by PPI, the goal of PPI was to ensure the relevance of the research with the lived community. In addition, by co-producing the research with those directly impacted by TS, the research aimed to be more impactful and better designed to suit the participants who would be involved. | Page 2 |
| --- | --- | --- |
| 2: Methods | The rationale and design of the study were informed by four PPI members, all of whom are co-authors on the paper, as well as input from the Tourette Syndrome Steering Group.  These members contributed to:   - Participant recruitment strategies - Reviewing and amending participant-facing documents (e.g., information sheets, consent forms, debrief sheets) - Co-designing focus group questions - Facilitating focus groups (PS co-facilitated all sessions) - Interpreting qualitative data - Co-authoring the final paper   They also played a central role in the dissemination strategy, including the co-production of an animation video with Woven Ink. | Page 2-3 |
| 3: Study results | PPI involvement led to:   - Greater access to participants via recruitment methods - Improved clarity and appropriateness of study materials for participants - Improved trust and authenticity from participants with the process - Empathetic and inclusive focus group discussion - More meaningful and rich data analysis | Page 4-6  Supplemental File 4 |
| 4: Discussion and conclusions | PPI involvement led to:   - Improved relevance of the research questions - Greater impact of non-academic dissemination including a co-produced animation video that reached over 500,000 people and received significant engagement on social media (2,500 likes, 1,000 shares) and increased visibility and advocacy for TS, including interest from a Member of Parliament | Page 3-4  Page 8  Supplemental File 4 |
| 5: Reflections/critical perspective | PPI had a substantial and positive influence throughout this research journey, from the rationale to its dissemination. Beyond the aforementioned impacts of including PPI in this project, involving PPI at this level is incredibly meaningful to the research team who find value in completing research alongside lived experience members and learn from this process.  The collaboration between academic and lived experience partners was central to the project’s success, demonstrating the value of inclusive research practices. | Page 3-4  Supplemental File 4 |
