# Supplementary material for: ‘For the love of God, just refer me’: a co-produced qualitative study of the experiences of people with Tourette Syndrome and tic disorders accessing healthcare services in the UK
Source: BMJ Open. 2025 Sep 5;15(9):e098306. doi: 10.1136/bmjopen-2024-098306 (PMC12414177; doi:10.1136/bmjopen-2024-098306)
Supplement: Supplementary file 5 [file bmjopen-2024-098306.reviewer_comments.pdf]

PEER REVIEW HISTORY

BMJ Open publishes all reviews undertaken for accepted manuscripts. Reviewers are asked to complete a checklist review form and are provided with free text boxes to elaborate on their assessment. These free text comments are reproduced below.

ARTICLE DETAILS

Title (Provisional)

“For the love of God, just refer me”: A co-produced qualitative study of the experiences of people with Tourette Syndrome and tic disorders accessing healthcare services in the UK

Authors

Babbage, Camilla May; Davies, E Bethan; Jones, Daniel P; Stevenson, Paul; Salvage, Jennifer; Anderson, Seonaid; McNally, Emma; Groom, Madeleine J

VERSION 1 - REVIEW

**Reviewer**	**1**
**Name**	**Shprecher, David**
**Affiliation**	**Cleo Roberts Center, Banner Sun Health Research Institute**
**Date**	**02-Apr-2025**
**COI**	**None**

In order to inform change to UK healthcare services for the chronic tic disorders population authors employed qualitative research using focus groups. Reflexive thematic analysis identified five primary themes characterizing the participants' experience navigating the healthcare system in the UK. General suggestions were made by the authors about the implications of these themes. These included holistic care (involving support at home, school and work) or multidisciplinary care, as well as implementation of education for clinicians about chronic tic disorders (as well as training on empathy).

The manuscript could potentially be improved in the following ways.

Themes 2, 3, and 5 all seem to emphasize the importance of a diagnosis, the challenge in accessing treatment, and concern that the healthcare system does not prioritize tics and Tourette’s. However, no mention was made in the discussion of the actual number of specialty centers that offer accurate diagnosis, treatment options, and prioritization of services for this population. Nor was mention made of the existing opportunities to facilitate referral and access to care- in particular broader availability of Telehealth in the post-COVID era. It would help to consider these in the discussion (and if the participants were not aware of these options, despite this information being just a Google search away for anyone with

access to a smartphone, then this should be discussed as a limitation to the research and an opportunity to mitigate the concerns raised above.

A minor correction to please consider- on page 5, lines 91-92, this sentence appears to be a fragmented missing the final words at the end of the phrase “for example survey data has previously. (25)”

**Reviewer**	**2**
**Name**	**Malli, Melina**
**Affiliation**	**University of Oxford, Oxford Institute of Population Ageing**
**Date**	**18-May-2025**
**COI**	**None**

One of the most commendable aspects of this study is its meaningful and sustained involvement of individuals with Tourette Syndrome at every stage of the research process—from shaping the research questions and designing the focus groups to co-facilitating data collection, interpreting findings, and supporting dissemination. This level of co-production remains rare in the literature on CTD/TS and positions the study as a pioneering example of participatory research in the field. The inclusion of reflexive statements and a lived experience facilitator enhances transparency and offers an important model for inclusive qualitative health research.

While the study offers valuable insights into the healthcare experiences of people with TS, there are areas where the analysis could have been further strengthened. Although five core themes are clearly articulated, the absence of sub-themes (with the exception of one inconsistently integrated example) limits analytical depth. Given the diversity of participant groups—adults, young people, and parents—sub-theming would have allowed for greater granularity and a more nuanced account of divergent or layered experiences.

In addition, the article would have benefited from more sustained engagement with relevant theoretical frameworks. Concepts from disability studies, neurodiversity paradigms, or the literature on structural stigma are notably absent, and their inclusion could have contextualised participants' accounts more robustly. The discussion also stops short of fully addressing the systemic causes of neglect, such as neurotypical bias, funding constraints, or policy inertia. While the emphasis on peer support, coping strategies, and resilience is valuable, there is a risk that this focus may unintentionally obscure institutional accountability or divert attention from the need for structural reform.

Finally, although the participatory design is a key strength, the article provides little critical reflection on how this approach shaped the research process and findings. It remains unclear whether co-production allowed for new insights to emerge, altered the interpretive lens, or influenced the thematic framework. Moreover, the study would be strengthened by

engagement with the broader literature on participatory or co-produced health research—particularly in relation to how lived experience informs methodological innovation and epistemic authority.

VERSION 1 - AUTHOR RESPONSE

Reviewer 1 (Dr. David Shprecher, Cleo Roberts Center, Banner Sun Health Research Institute) - Comments to the Author:

In order to inform change to UK healthcare services for the chronic tic disorders population authors employed qualitative research using focus groups. Reflexive thematic analysis identified five primary themes characterizing the participants' experience navigating the healthcare system in the UK. General suggestions were made by the authors about the implications of these themes. These included holistic care (involving support at home, school and work) or multidisciplinary care, as well as implementation of education for clinicians about chronic tic disorders (as well as training on empathy).

The manuscript could potentially be improved in the following ways.

COMMENT: Themes 2, 3, and 5 all seem to emphasize the importance of a diagnosis, the challenge in accessing treatment, and concern that the healthcare system does not prioritize tics and Tourette's. However, no mention was made in the discussion of the actual number of specialty centers that offer accurate diagnosis, treatment options, and prioritization of services for this population. Nor was mention made of the existing opportunities to facilitate referral and access to care- in particular broader availability of Telehealth in the post-COVID era. It would help to consider these in the discussion (and if the participants were not aware of these options, despite this information being just a Google search away for anyone with access to a smartphone, then this should be discussed as a limitation to the research and an opportunity to mitigate the concerns raised above.

Thank you for highlighting this important issue. We welcome the opportunity to provide further information about healthcare services for tics in England. Since submitting this manuscript for review, we have had another paper accepted for publication (Rattu et al., 2025). In this paper, we report findings from a mixed-methods study which included a survey of service provision in England. The survey showed that there are only 12 providers of a tic service for children and young people across the whole of England (from a sample of 234 care providers). This demonstrates the lack of provision for the referral, assessment and management of tics in this age group in England. What was also interesting (although not included in the paper) is that the provider of tic services in each locality within England was unclear in many cases, with no named provider. We initially tried searching online to find this information but given the complexity in healthcare service organisation in England, and the lack of services for tics, this was not fruitful.

In the same paper, we also present findings from a national survey of healthcare professionals working in services for children and young people. They reported experiencing a lack of training, support and resources to support them in assessing and treating tics, and a lack of expertise across the country. Although we did not ask specifically about telehealth within the surveys in this study, since very few services accept referrals for tic disorders, it seems unlikely that they offer telehealth for this population either.

Finally, the National Institute for Health and Care Excellence (NICE) in the UK recently conducted a survey across all age groups of people with tics (or their parents if under 16 years of age). They reported that of 1508 respondents almost 25% waited over 3 years for a diagnostic assessment. Of those with a diagnosis, over 60% had not been offered any treatment for their tics.

At the time of designing, conducting and analysing the focus groups presented in this manuscript, we did not have access to any of this information. Now that the findings of our paper (in which we also cite the NICE survey) have been accepted for publication, we have provided a brief summary in the discussion section of this paper on Pages 18-19, starting "*Findings from a recent mixed-methods study have identified only twelve care boards within the UK have a clinical service pathway for assessment and treatment of tics, with the majority of these found within London (19)*". We feel this provides useful context for readers who are not based in the UK, or who are less aware of the severe shortage of healthcare provision for tic disorders in the UK currently.

COMMENT: A minor correction to please consider- on page 5, lines 91-92, this sentence appears to be a fragmented missing the final words at the end of the phrase "for example survey data has previously. (25)"

Thank you for pointing this out. The phrase has now been deleted as part of broader changes to that section of the introduction.

Reviewer 2 (Dr. Melina Malli, University of Oxford) - Comments to the Author:

One of the most commendable aspects of this study is its meaningful and sustained involvement of individuals with Tourette Syndrome at every stage of the research process—from shaping the research questions and designing the focus groups to co-facilitating data collection, interpreting findings, and supporting dissemination. This level of co-production remains rare in the literature on CTD/TS and positions the study as a pioneering example of participatory research in the field. The inclusion of reflexive statements and a lived experience facilitator enhances transparency and offers an important model for inclusive qualitative health research.

We thank the reviewer for their positive comments about our study and their understanding of the efforts required to complete co-production at this level.

COMMENT: While the study offers valuable insights into the healthcare experiences of people with TS, there are areas where the analysis could have been further strengthened. Although five core themes are clearly articulated, the absence of sub-themes (with the exception of one inconsistently integrated example) limits analytical depth. Given the diversity of participant groups—adults, young people, and parents—sub-theming would have allowed for greater granularity and a more nuanced account of divergent or layered experiences.

Thank you to the reviewer for spending the time reviewing the themes and offering this critical reflection. The authors have re-reviewed the themes, including taking a close look at the coding and structure of the themes, to ensure that there are no missing sub-themes. Having done this, we remain confident that no further sub-themes are necessary, following guidelines from Braun & Clarke that "s*ubthemes generally should be used sparingly, only when there is one particular element of a theme that has a particular focus, is notable, and/or is particularly important for the*

research question" (*https://cdn.auckland.ac.nz/assets/psych/about/our-research/documents/Answers%20to%20frequently%20asked%20questions%20about%20thematic%20analysis%20April%202019.pdf*). Nonetheless, we recognise that the way the themes are presented may lead them to appearing superficial or looking like domain summaries. To overcome this, we have spent time reviewing the names of themes and we have highlighted more clearly where certain themes were more prevalent within certain population groups. Given the similarity in experiences across the sample, we did not feel enough nuance within the groups for this to be conceptualised as a sub-theme and neither was it deemed necessary to answer this research question which aims to look across children, young people, and adults' experiences together. We hope this gives a better account of their experiences within the overall theme.

COMMENT: In addition, the article would have benefited from more sustained engagement with relevant theoretical frameworks. Concepts from disability studies, neurodiversity paradigms, or the literature on structural stigma are notably absent, and their inclusion could have contextualised participants' accounts more robustly. The discussion also stops short of fully addressing the systemic causes of neglect, such as neurotypical bias, funding constraints, or policy inertia. While the emphasis on peer support, coping strategies, and resilience is valuable, there is a risk that this focus may unintentionally obscure institutional accountability or divert attention from the need for structural reform.

Thank you for this comment – which has helped the authors to acknowledge that a multidisciplinary and multi-layered approach is needed in comprehending this issue further. We have now added some text to the discussion section to place the study and its findings within these broader models and contexts, bearing in mind the word limit for this journal.

COMMENT: Finally, although the participatory design is a key strength, the article provides little critical reflection on how this approach shaped the research process and findings. It remains unclear whether co-production allowed for new insights to emerge, altered the interpretive lens, or influenced the thematic framework. Moreover, the study would be strengthened by engagement with the broader literature on participatory or co-produced health research—particularly in relation to how lived experience informs methodological innovation and epistemic authority.

This is a valid comment, which the authors appreciate to improve the manuscript. To ensure the coproduction processes are adequately reported in this manuscript, the GRIPP2 reporting checklist for PPI has been included as an Additional File (4). Further, justification for including PPI in terms of leading to improved healthcare has been added to the introduction on Pages 5-6, Lines 110-121.

VERSION 2 - REVIEW

**Reviewer**	**1**
**Name**	**Shprecher, David**
**Affiliation**	**Cleo Roberts Center, Banner Sun Health Research Institute**
**Date**	**13-Jul-2025**

COI

This authors concerns/recommendations were addressed for the most part, however in the discussion section the authors did not seem familiar with the use of telemedicine (aka telehealth) to expand access to specialist consultations (as opposed to just CBIT specialists). The following reference is one of many that points out how even at the start of the COVID pandemic, access to movement disorders specialist care around the word as markedly increased- Hassan A, Mari Z, Gatto EM, Cardozo A, Youn J, Okubadejo N, Bajwa JA, Shalash A, Fujioka S, Aldaajani Z, Cubo E; International Telemedicine Study Group. Global Survey on Telemedicine Utilization for Movement Disorders During the COVID-19 Pandemic. Mov Disord. 2020 Oct;35(10):1701-1711. doi: 10.1002/mds.28284. PMID: 32833273; PMCID: PMC7461376.

If, in the modern day Post COVID era this is still not being offered by Tourette programs in the southern UK then it probably should be (and the authors would be reemiss in not mentioning it).

Pls note Reference 46 appears to be incomplete (does not list the journal/book or publication year), just the title. The manuscript should not be published without completing this or any other errors in the references.

**Reviewer**	**2**
**Name**	**Malli, Melina**
**Affiliation**	**University of Oxford, Oxford Institute of Population Ageing**
**Date**	**13-Jul-2025**
**COI**	

Excellent paper.

VERSION 2 - AUTHOR RESPONSE

Reviewer 1 (Dr. David Shprecher, Cleo Roberts Center, Banner Sun Health Research Institute) - Comments to the Author:

Comments to the Author:

This authors concerns/recommendations were addressed for the most part, however in the discussion section the authors did not seem familiar with the use of telemedicine (aka telehealth) to expand access to specialist consultations (as opposed to just CBIT specialists). The following reference is one of many that points out how even at the start of the COVID pandemic, access to movement disorders specialist care around the word as markedly increased- Hassan A, Mari Z, Gatto EM, Cardozo A, Youn J, Okubadejo N, Bajwa

JA, Shalash A, Fujioka S, Aldaajani Z, Cubo E; International Telemedicine Study Group. Global Survey on Telemedicine Utilization for Movement Disorders During the COVID-19 Pandemic. Mov Disord. 2020 Oct;35(10):1701-1711. doi: 10.1002/mds.28284. PMID: 32833273; PMCID: PMC7461376.

If, in the modern day Post COVID era this is still not being offered by Tourette programs in the southern UK then it probably should be (and the authors would be reemiss in not mentioning it).

Thank you to Reviewer 1 for reviewing the previous edits. We are pleased you felt that the concerns and recommendations had been appropriately handled and we thank you again for your time in supporting us with revising this manuscript.

We agree with Reviewer 1's comment that telemedicine could be better utilised to increase access to specialist services, especially since Covid where we are aware that the use of such platforms has increased availability of such services. We have now added the term 'telehealth' (page 19, line 471) to identify this as a potential option alongside 'videoconferencing' to improve healthcare access and treatment in the UK. We have further expanded this point by adding a sentence (Page 19, lines 472-474) to highlight the lack of routine use of these methods within UK services "Freedom of Information requests suggest such methods are not currently used routinely in the UK for tic disorders [19]".

Pls note Reference 46 appears to be incomplete (does not list the journal/book or publication year), just the title. The manuscript should not be published without completing this or any other errors in the references.

Thank you for carefully reviewing such details in the manuscript. We have reviewed reference 46 (now Ref 47) and all others references to ensure they are complete and correct.

Reviewer 2 (Dr. Melina Malli, University of Oxford)

Comments to the Author:

Thanks very much to Reviewer 2 for their kind words. We'd also like to extend our thanks to Reviewer 2 for their previous considered comments, which we feel have substantially improved this manuscript.
